# Tuning magnetic properties of penta-graphene bilayers through doping with boron, nitrogen, and oxygen

**DOI:** 10.1038/s41598-020-73901-8

**Published:** 2020-10-07

**Authors:** Ramiro Marcelo dos Santos, Wiliam Ferreira da Cunha, Rafael Timóteo de Sousa Junior, William Ferreira Giozza, Luiz Antonio Ribeiro Junior

**Affiliations:** 1 Institute of Physics, University of Brasília, 70919-970 Brasília, Brazil; 2 Department of Electrical Engineering, University of Brasília, Brasília, 70919-970 Brazil

**Keywords:** Materials science, Materials for devices, Theory and computation

## Abstract

Penta-graphene (PG) is a carbon allotrope that has recently attracted the attention of the materials science community due to its interesting properties for renewable energy applications. Although unstable in its pure form, it has been shown that functionalization may stabilize its structure. A question that arises is whether its outstanding electronic properties could also be further improved using such a procedure. As PG bilayers present both sp$^2$ and sp$^3$ carbon planes, it consists of a flexible candidate for functionalization tuning of electromagnetic properties. In this work, we perform density functional theory calculations to investigate how the electronic and structural properties of PG bilayers can be tuned as a result of substitutional doping. Specifically, we observed the emergence of different magnetic properties when boron and nitrogen were used as dopant species. On the other hand, in the case of doping with oxygen, the rupture of bonds in the sp$^2$ planes has not induced a magnetic moment in the material.

## Introduction

The interest in two-dimensional extended nanostructures was further highlighted after the graphene arising^1,2^. The properties of graphene are truly unique^3^, and a wide range of applications have been predicted for this carbon allotrope^4^. Although the overall success of graphene, there are still some limitations to be overcome regarding its optoelectronic applications, the main being its null bandgap^5^. For that reason, other two-dimensional materials have been extensively tested. Among them, transition metal dichalcogenides (TMDs)^6–8^ and novel graphene-based allotropes^9–11^ stand out. However, graphene allotropes still hold an important advantage over TMDs, for instance, of being formed only by carbon atoms, which makes it easier to synthesize on a large scale. Therefore, it would be ideal to conceive a material that, while presenting semiconducting properties would still be based on graphene.

Having such a goal in mind, several theoretical and experimental studies have been conducted to achieve alternative solutions to monolayer graphene^9–17^. For instance, studies concerning graphene bilayer concluded that its structural properties are altered from a suitable layer stacking engineering^18–20^. In one of these studies, it was obtained that the rotation of one graphene layer over another one leads to the displacement of the Dirac cones, but no gap opening was observed^18^. The conclusion is that layer stacking still need to be associated with other procedure if a graphene-based system with semiconducting properties is to arise. So far, the main option relied on substitutional doping^21–24^ on graphene structure. Density functional theory (DFT) calculations were performed to study the doping of boron (B) and nitrogen (N) in graphene bilayers^25^. The results revealed that for doping with B (N), a gap opening from 0.11 to 0.32 eV (0.09 and 0.30 eV) was obtained, being these values depend on the type of bilayers packing^25^.

Another approach is to study other possible arrangements: different, and yet based on graphene. Among them are carbon nanotubes^26^, nanoribbons^27^, nanoscrolls^28^, and novel graphene-based allotropes, such as popgraphene^11^, phagraphene^10^, and penta-graphene^9^ (PG). In its pure form, the PG structure is meta-stable but presents an almost direct bandgap $\sim 2.4$ eV, which is interesting for optoelectronic applications. Its structure is similar to the pentagonal tiles of Cairo, where the carbon atoms occupy three types of planes: a plane of sp$^3$ carbons sandwiched by two planes of sp$^2$ carbons^9^. Such a structure allows more degrees of freedom for doping when contrasted to graphene. As a result, different ways of modulating its electronic properties can be obtained^29^. Particularly, it can be reasoned that the flexibility of such material makes it a promising candidate to fulfill the role of the carbon allotrope with semiconducting-like properties. This fact is especially true if one considers the two promising factors on PG: considering bilayers and substitutional doping.

It is well known that the PG bandgap increases with an increase in oxygen doping concentration^30–32^. Such a trend is attributed to the up-shift of the conduction band minimum during the oxidation process^30^. Since oxidized PG exhibits a large bandgap, it can be considered a good alternative for the conception of new dielectric layers in electronic devices^31^. When it comes to doped carbon-based nanostructures, boron and nitrogen are the most often used dopants once their similar electronic structure and size to carbon allow their incorporation into the carbon-based substrate with minimal strain and changes in the lattice arrangement^33–38^. Moreover, the boron- and nitrogen-doped PG can effectively decompose H2 molecules into two H atoms, which is an interesting feature for energy conversion and storage applications^36,39^. In this sense, oxygen-, nitrogen-, and boron-doped PG systems must be further studied to propose new routes in developing PG-based devices.

In the present work, we explored the structural and electronic properties of doped PG bilayers by carrying out state of art electronic structure calculations on the DFT level of theory. This study was motivated by the fact that PG has more degrees of freedom to modulate its electronic properties than graphene. We used as substitutional dopants boron, nitrogen, and oxygen atoms. It was observed that the introduction of oxygen atoms in the sp$^2$ plane leads to the breaking of bonds and subsequently no magnetic properties take place. Remarkably, boron and nitrogen doping in the sp$^3$ plane, in turn, gives rise to a significant magnetization which was not observed for the cases in which this dopant was introduced in the sp$^2$ plane. As mentioned above, PG packing allows us to have extra degrees of freedom for doping. This feature is interesting since in PG monolayers there are two sp$^2$ planes that can interact with the external environment. This feature, in turn, creates another degree of freedom for doping and, consequently, for modulation of its electronic properties.

## Results

**Figure 1:**
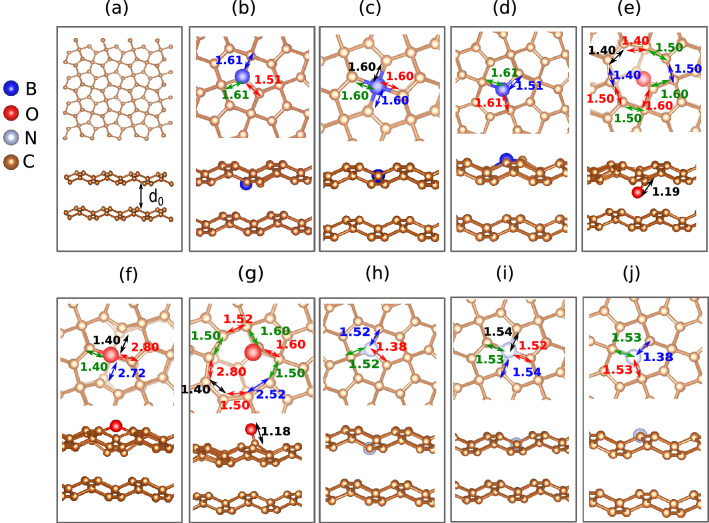
Schematic representation of the lattice arrangement (structural properties) for optimized PG bilayers doped with boron (**b**–**d**), oxygen (**e**–**g**), and nitrogen (**h**–**j**). The radius of the spheres that represent the dopant atoms was increased for better visualization of the dopant sites.

**Figure 2:**
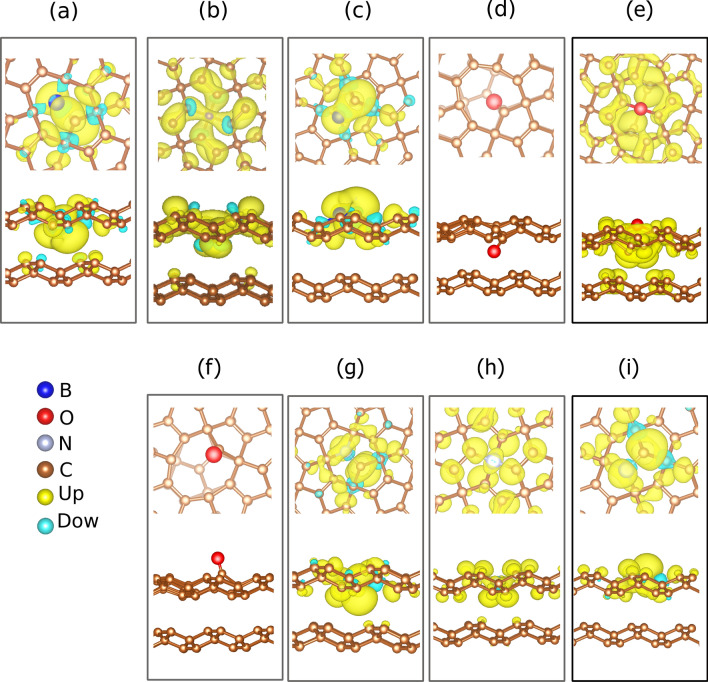
Charge density distribution, $\rho (Up)-\rho (Down)$, obtained by adopting an isovalue of 0.001. The radius of the spheres that represent the dopant atoms was increased for better visualization of the dopant sites.

We begin our discussions by presenting the structural properties (Fig. 1) and the charge localization (Fig. 2) profiles for the model doped PG bilayers studied here. In these figures, the radius of the spheres that represent the dopant atoms was increased for better visualization of the dopant sites. Figure 1a presents the optimized geometry of the two layers without defects, i.e. before the doping procedure took place. Figure 1b–d represent the substitutional doping with boron, Fig. 1e–g illustrate the substitutional doping with oxygen, and Fig. 1h–j account for the nitrogen-based functionalization. The PG layers were initially packed with 4.0 Å of the distance between them by matching their rings to compose the bilayer complexes. Subsequently, these complexes were optimized to obtain their ground state solutions. The results presented in Fig. 1 (structural properties) do not show any relative displacement between the two layers upon doping. In the doping mechanism adopted here, the PG plane at the bottom is always pristine. The second PG layer on top receives the dopant in three distinct channels: (dopant-sp$^2$-out) when the dopant is placed in the $sp^2$ plane above the interlayer region interacting with the vacuum, (dopant-sp$^2$-in) when the dopant is placed in the $sp^2$ plane within the interlayer region interacting with the PG plane at the bottom, and (dopant-sp$^3$) for the doping in the sp$^3$ plane. For these nomenclatures, “dopant” stands for oxygen (O), boron (B), or nitrogen (N) atom. Importantly, some theoretical works have studied the structural stability of oxygen-, nitrogen-, and boron-doped PG layers^29,29,33,36,39,39–41,41^. In a reactive molecular dynamics study, the results have revealed that oxygen-doped PG layers present remarkable enhancement in failure stress and strain when contrasted with pristine PG layers^40^. Moreover, it was recently demonstrated, by using DFT calculations, that boron- and nitrogen-doped PG layers are structurally stable when concentrated with pristine PG layers^29,36,39^.

Figure 1b–d illustrate the cases B-sp$^2$-in, B-sp$^3$, and B-sp$^2$-out, respectively. One can observe that the bond lengths between *B*–*C* slightly deviate from that of the bond length *C*–*C* in the pristine monolayer, which is 1.57 Å. These bond lengths (*B*–*C*) assume minimum and maximum values of 1.51–1.61 Å for all the cases of doping with B. As for the oxygen doping picture, represented by Fig. 1e–g, we observe a greater deformation around the doping site. Figure 1e,g show the result of doping the sp$^2$ planes. It is possible to note a tendency of carbonyl formation with the elevation of the oxygen atom by the distance of 1.18 and 1.19 Å from the carbon atom in the respective sp$^3$ plan. With the elevation of the oxygen from the sp$^2$ plane, the formation of a vacancy is observed with bond lengths in the edges whose sizes vary from 1.40 to 1.60 Å for the case O-sp2-in and O-sp2-out. In the case O-sp3 (Fig. 1f), a tendency of the oxygen to leave the plane is also observed, as the bond length of 1.40–1.72 Å is achieved between the first neighbors of the dopant. The nitrogen-doped PG lattices, Fig. 1h–j, present similar results to the ones for the boron-doped case. The bond lengths between *N*–*C* also slightly deviate from the C–C ones in the pristine case. These bond lengths (*N*–*C*) assume minimum and maximum values of 1.38–1.52 Å for all doping channels.

The charge density wrapped around the dopant is depicted in Fig. 2. The yellowish cloud stands for the density due to up spin electrons, whereas the blueish on of the down spin electrons. The net charge observed is responsible for the magnetic moment, which characterizes a magnetization due to the presence of the dopant. The values of the magnetic moment for each structure are listed in Table 1, to be discussed later. It is observed that this charge concentration is more effective in the case of doping with boron (Fig. 2a–c) and nitrogen (Fig. 2g–i). On the other hand, no spontaneous magnetization was observed in the O-sp2-in and O-sp2-out cases (Fig. 2d–f). This behavior suggests that magnetization in the case of doping with oxygen is not a direct consequence of doping, but rather of the deformation of the geometry that the dopant produces. The sp$^3$ plane has two dangling carbons with a distance of 2.80 and 2.72 Å from the oxygen atom. This distance makes the $\pi$-electrons of these two atoms to contribute to the effective magnetization of the bilayer, whose magnetic moment is 2.00 Bohr Magneton. In the O-sp2-in and O-sp2-out cases, the non-concentration of charge in the oxygen atom, which makes two connections in the plane, suggests a double bonding with the carbon, which characterizes a carboxyl. The resulting bond configuration for the carbon atoms in the edge keeps the symmetry of the vacancy.

The first row of Table 1 presents the formation energy, which is defined as: $E_{form} = E_b-(E_{mp}+E_{md})$, where $E_b$ is the total energy for each doped bilayer, $E_{mp}$ the total energy of the monolayer without defect, and E$_{md}$ total energy of the doped bilayer. One can observe that the energy cost (average value) for the formation of the bilayer is approximately the same for boron and oxygen doping, i.e., − 9.49 to − 9.53 eV, respectively. For the nitrogen doping case, the average value of the formation energy is − 7.54 eV. In the second row of this table, we present the cohesion energy that is given by $E_{coh} = \frac{E_{total}-(E_{C}N_{C}+E_{X}N_{x})}{N_{total}}$, where $E_{C}$ is the total energy of a carbon atom and $N_{C}$ is the number of carbon atoms of the bilayer. $E_{x}$ and $N_{x}$ are the total energies and number of doping atoms respectively, where *X* stands for B, N, or O. $N_{total}$ is the total number of atoms in the bilayers. The cohesion energy has values similar for all systems, suggesting the same level of cohesion for the three types of dopants. In the third row, we present the magnetic moment of each bilayer. One can realize that for the O-sp2 case, the value of the magnetic moment is zero, which is in agreement with what was already discussed of the net charge density. In the fourth row, the distances $d_0$ between the two layers are shown. After optimizing the geometry of the bilayers, these distances remained larger for the two both B-sp2-in and O-sp2-in doping cases, compared to the others that were between 2.50 and 2.61 Å, while for B-sp2-in and O-sp2-in is 3.12 and 2.70 Å, respectively. These results suggest that the dopant has a significant contribution to the interactions between the two layers.

**Table 1:** Formation energy, cohesion energy, magnetic moment and distance between the two layers for all the cases studied here.

	without dopant	B-sp2-in	B-sp3	B-sp2-out	O-sp2-in	O-sp3	O-sp2-out	N-sp2-in	N-sp3	N-sp2-out
E$_{form}$ (eV)	$-$ 10.20	$-$ 9.54	$-$ 9.55	$-$ 9.39	9.60	$-$ 9.59	$-$ 9.41	$-$ 7.51	$-$ 7.60	$-$ 7.52
E$_{coh}$ (eV)	$-$ 7.88	$-$ 8.38	$-$ 8.37	$-$ 8.37	$-$ 8.42	$-$ 8.48	$-$ 8.42	$-$ 8.15	$-$ 8.14	$-$ 8.15
|**m**| ($$\mu _B$$)	0.0	1.00	0.93	1.00	0.00	2.00	0.00	1.00	0.92	1.00
d$_0$ (Å)	2.95	3.12	2.50	2.60	2.70	2.60	2.60	2.61	2.57	2.61

**Figure 3:**
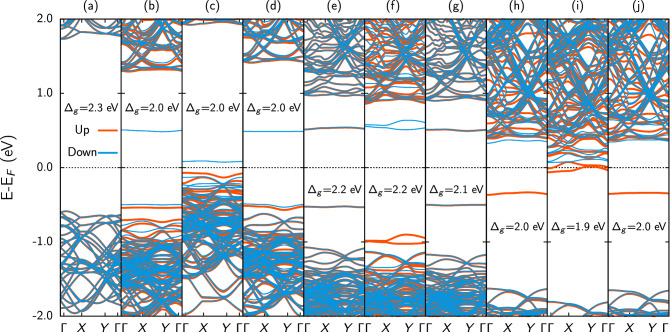
Band structure for the doped PG bilayers studied here. (**a**) Pristine structure, (**b**) B-sp2-in, (**c**) B-sp3, (**d**) B-sp2-out, (**e**) O-sp2-in, (**f**) O-sp3, (**g**) O-sp2-out, (**h**) N-sp2-in, (**i**) N-sp3, and (**j**) N-sp2-out.

Figure 3 presents the band structure of the doped bilayer bands. For the pure bilayer (Fig. 3a), one can note that an indirect gap of 2.3 eV takes place, a value approximately equal to that found for the PG monolayer^9^. In the case of doping with boron (Fig. 3b–d) and nitrogen (Fig. 3h–j), we observe a reduction of the bandgap to approximately 2.0 eV, which remained indirect. For the two cases of sp$^2$ doping (Fig. 3b,d,h,j), it occurs the emergence of states in the middle of the bandgap. In Fig. 3b,d, one can note a downstate above the Fermi level, one up and other down states, below the Fermi level. On the other hand, in Fig. 3h,j an upstate below the Fermi level. For the sp$^3$ doping cases (Fig. 3c,f,i), the two states are symmetrically positioned with respect to the Fermi level. In the case of oxygen doping, we also observed the appearance of the states in the middle of the bandgap: these states are symmetrical concerning the Fermi level and have no spin degeneration for the O-sp2-in and O-sp2-out cases (Fig. 3e,g). In these configurations, the bandgap was reduced to 2.2 and 2.1 eV, respectively. For the O-sp3 case (Fig. 3f), the bandgap is also 2.2 eV and with the two symmetrical states about the Fermi level, with spin up below and down above the Fermi Level.

**Figure 4:**
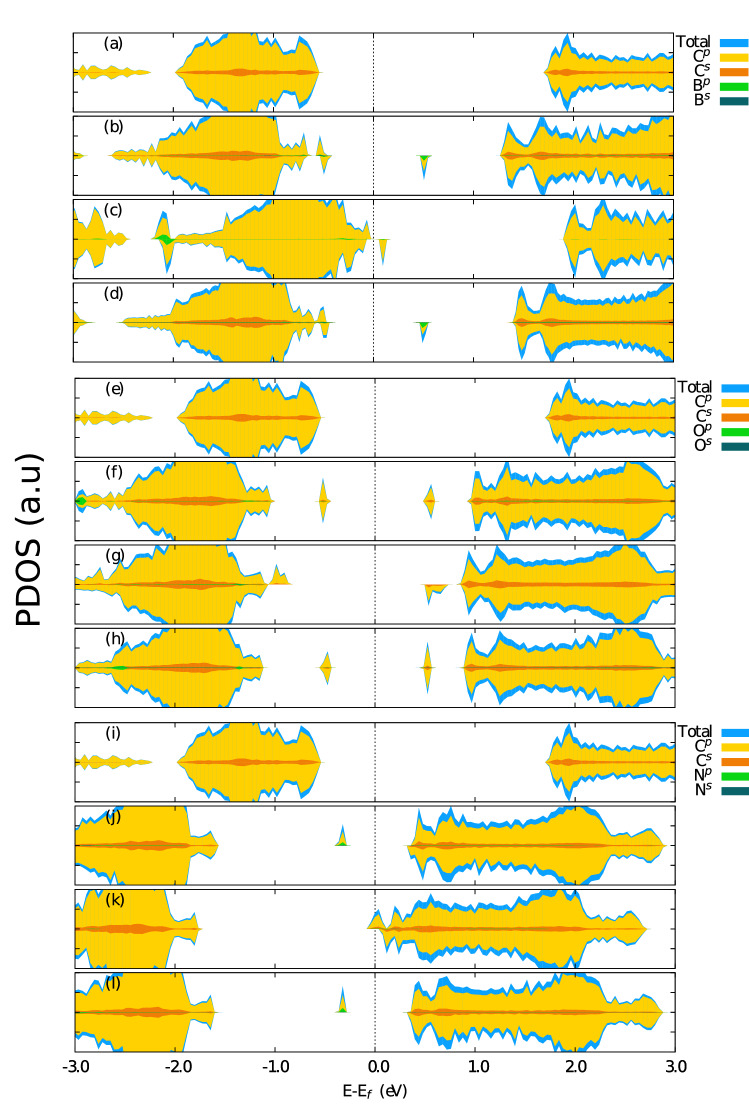
Projected density of states (PDOS) for the doped PG bilayers studied here. (**a**) Pristine structure (**b**) B-sp2-in (**c**) B-sp3 (**d**) B-sp2-out, (**e**) O-sp2-in, (**f**) O-sp3, (**g**) O-sp2-out, (h) N-sp2-in, (**i**) N-sp3, and (**j**) N-sp2-out.

Finally, Fig. 4 presents the projected density of states (PDOS) for all the complexes studied here. For the sake of comparison, Fig. 4a,e,i present the PDOS for the pristine case. For all the cases, the most significant contribution to the formation of the bands is for the *p* states of $sp^2$ carbon atoms. The Fermi level is closer to the valence bands, which characterizes a *n*-type semiconductor. In the B-sp$^2$ cases (Fig. 4b,d), a slight contribution of *p* orbitals of boron at the first peak in the middle of the bandgap (above the Fermi level) is observed. Still, for these two cases, we observed that the Fermi level is closer to the valence band, which characterizes a *n*-type semiconductor. The N-sp$^2$ cases (Fig. 4j,l) present a similar trend for PDOS when compared to the B-sp$^2$ cases. Regarding the B-sp$^3$ and N-sp$^3$ cases (Fig. 4c,k, respectively), there is no significant contribution of the dopants to the states near the Fermi level and the structure behaves as a *n*-type semiconductor, once the Fermi level is touching the top of the valence band. In the case of oxygen doping (Fig. 3f–h), we did not observe any significant contribution from O atoms. In the O-sp2-in case (Fig. 3f), the Fermi level is in the middle of the bandgap with peaks symmetrically localized regarding it for both spin channels, which characterizes the non-magnetization of this material. In the O-sp3 case (Fig. 3g), we have observed that the Fermi level is slightly displaced towards the conduction states, which characterizes a *p*-type semiconductor. It is also observed antisymmetric peaks concerning the spin, which stands for a significant magnetic moment of this structure. For the O-sp2-out case (Fig. 3h), a slight displacement of the Fermi level near the conduction band was also observed, characterizing a *p*-type semiconductor. It is observed that the peaks closest to the Fermi level are symmetrical concerning the spin states, proving the non-magnetic behavior of the material.

## Methods

To investigate the electronic structure of doped PG bilayers, we used DFT calculations as implemented in the SIESTA software^42,43^. It makes use of a numerical base to expand the wave functions of the many atoms system. In the present work, it was used the DZP basis set^44,45^. As for the functional approximation, it was considered the generalized approximation of the gradient proposed by Perdew, Burke, and Ernzerhof (GGA/PBE) + vDW^46^, which is built from the expansion of the second-order density gradient. Pseudopotentials parameterized within the Troullier-Martins formalism are also considered^47^. This approximation is of fundamental importance for the description of the magnetic and electronic properties of materials composed of atoms with many electrons. All calculations were performed considering spin polarization. To calculate the bands and state densities, an MPK mesh of 15 $\times$ 15 $\times$ 1 is used^48^. A mesh cut of 200 Ry is chosen as a parameter for our calculations^49^. The forces converged until reaching a minimum value of 0.001 eV/Å. In order to ensure a good compromise between the accuracy of our results and the computational feasibility, the tolerance in the density of the matrix and the total energy was set at 0.0001 and 0.00001 eV, respectively. Importantly, this set of parameters were used recently to study other carbon-based lattices^29,50–52^.

## Conclusion

In summary, we carried out DFT calculations to investigate the influence of boron, nitrogen, and oxygen doping on the electronic properties of PG bilayers. Our findings showed that the difference between dopant on the sp$^2$ and sp$^3$ planes have a significant impact on the magnetic properties of boron and nitrogen doping. It was observed a spontaneous magnetization in the system when these doping species were considered. This is because boron and nitrogen contains one and three electrons, respectively, in the orbital valence 2*p*, whereas the substituted carbon has a pair of electrons in this same orbital. Therefore, the *C*–*B* and *C*–*N* bonds result in a magnetic polarization due to electronic covalence. This effect is characterized by the flat energy levels that appear in the middle of the bandgap for the cases of boron and nitrogen doping in the sp$^2$ planes. Such an effect is not observed in the case of doping with oxygen due to bond breaking. Regarding the effect on the pristine layer, there was no significant difference in its electronic properties. In the cases of boron and nitrogen doping, the resulting system characterizes a *n*-type semiconductor. On the other hand, for the oxygen doping in the sp$^3$ plane, we have observed that the Fermi level is slightly displaced towards the conduction states, which characterizes a *p*-type semiconductor. Since the electronic structure of PG bilayers present extra doping channels and can be easily tuned by doping its structure with just a single atom, they can represent an interesting alternative for replacing graphene in some optoelectronic applications.
